# Metabolic checkpoints in bone remodeling: from dysregulation to targeted therapies

**DOI:** 10.1016/j.clinsp.2026.101060

**Published:** 2026-07-29

**Authors:** Xuequan Zhao, Xixi Han, Baoguo Wang, Wenjie Gao, Zhongyong Zhang

**Affiliations:** a Department of Orthopedics, Cangzhou Clinical College of Integrated Traditional Chinese and Western Medicine of Hebei Medical University (Hebei Key Laboratory of Integrated Traditional and Western Medicine in Osteoarthrosis Research), Cangzhou, China; b Department of Graduate School, Nanjing University of Chinese Medicine, Nanjing, China; c Department of Encephalopathy, Second Affiliated Hospital of Anhui University of Chinese Medicine, Hefei, China; d Department of Graduate School, Soochow University, Suzhou, China

**Keywords:** Bone remodeling, Metabolic reprogramming, Osteoblast, Osteoclast, Warburg effect, RANKL/RANK/OPG pathway, Targeted therapy

## Abstract

Bone remodeling depends on a tightly regulated metabolic crosstalk between osteoblasts and osteoclasts. The article advances a hypothesis‑driven framework that positions discrete metabolic checkpoints ‒ glycolytic flux, mitochondrial Oxidative Phosphorylation (OXPHOS), amino‑acid utilization, and ion‑channel-coupled calcium signaling ‒ as tractable levers for restoring formation-resorption balance. The authors explicitly examine metabolic heterogeneity within and across osteolineages and disease contexts, integrate immune-metabolic coupling that links TCA‑cycle intermediates to RANKL/OPG regulation, and synthesize evidence on the efficacy and trade‑offs of current and emerging therapies (e.g., sclerostin/Wnt restoration, RANKL blockade, HIF‑1α modulation, and calcium‑channel targeting). To avoid over‑interpretation, clinical statements are constrained to the strength of available evidence; preclinical findings are labeled accordingly. The authors further propose a pragmatic framework for precision modulation ‒ rather than blanket inhibition ‒ of metabolic pathways to minimize off‑target toxicity and enable context‑dependent benefit. This consolidated view reframes metabolic dysfunction as a driver of skeletal pathology and a blueprint for targeted interventions.

## Highlights

• Metabolic Checkpoint: nutrient stress shifts bone from regeneration to resorption.
• Glycolysis supports osteoblasts yet drives metastasis via lactylation.
• Osteoblast metabolic heterogeneity shapes vulnerabilities and therapeutic windows.
• Balance efficacy and toxicity of HIF-1α inhibitors and sclerostin mAbs.

## Introduction

Cell metabolism underpins the normal physiological functions of cells. All tissues and organs in the body derive their energy through cellular metabolic processes, during which substantial amounts of fats and proteins are consumed.^1^ Multiple mechanisms of energy and substrate metabolism have been identified, among which the direct consumption of glucose and fatty acids is most common.^2^ In animal experiments, glucose or its aqueous solution is often introduced into the cell culture medium to stimulate osteoblasts. In human studies, biological materials such as cholesterol, triglycerides, phospholipids, and sphingolipids are frequently used to stimulate osteoblasts.^3^ Additionally, adipose tissue has been shown to promote osteogenesis by stimulating the synthesis and secretion of bone matrix components and by directly consuming amino acids.

Beyond the direct consumption of energy and substrates, cellular metabolism also affects bone formation and maintenance through influences on mitochondrial function,^4^ ribosomal function,^5^ and the expression of various proteins.^6^ Osteoblasts and osteoclasts are the primary representative bone cells. This review summarizes key studies on osteoblasts and osteoclasts, along with the associated metabolic pathways and mechanisms, from the perspective of cell metabolism. The authors also discuss the classifications of cellular metabolism relevant to bone and cartilage regeneration, bone homeostasis, and metabolic bone diseases.

Despite established roles of glycolysis, oxidative phosphorylation, and Wnt/NF-κB in osteoblast metabolism, fundamental gaps persist. For instance, metabolic heterogeneity among osteoblast subtypes (e.g., matrix-synthesizing vs. mineralizing osteoblasts) remains uncharacterized, and its implications for bone diseases like osteoporosis are unexplored. Further, conflicting evidence on mTORC1’s dual anabolic/catabolic roles in bone formation warrants systematic reevaluation. The work adheres to the PRISMA guidelines, and no new human or animal research was conducted.

## Cellular metabolism in relation to osteoblasts

Within bone cells, cellular metabolism can modulate bone formation through processes such as the synthesis and breakdown of substances, ATP production, and enzymatic regulation.^7^ For instance, osteoblasts directly consume glucose, fatty acids, or amino acids to synthesize enzymes and small RNAs, and also maintain intracellular ATP levels and enzyme activity. Two major protein synthesis pathways in osteoblasts are NFκB^8^ and Wnt.^9^ NFκB is critical for osteoblast differentiation, proliferation, and mineralization, whereas Wnt/β-catenin signaling regulates osteoblast proliferation and differentiation, in turn affecting other intracellular proteins.^10^ The interaction between Wnt/β-catenin and NFκB is important not only in tumorigenesis but also has potential applications in regulating bone inflammation.^11^

**Fig. 1:**
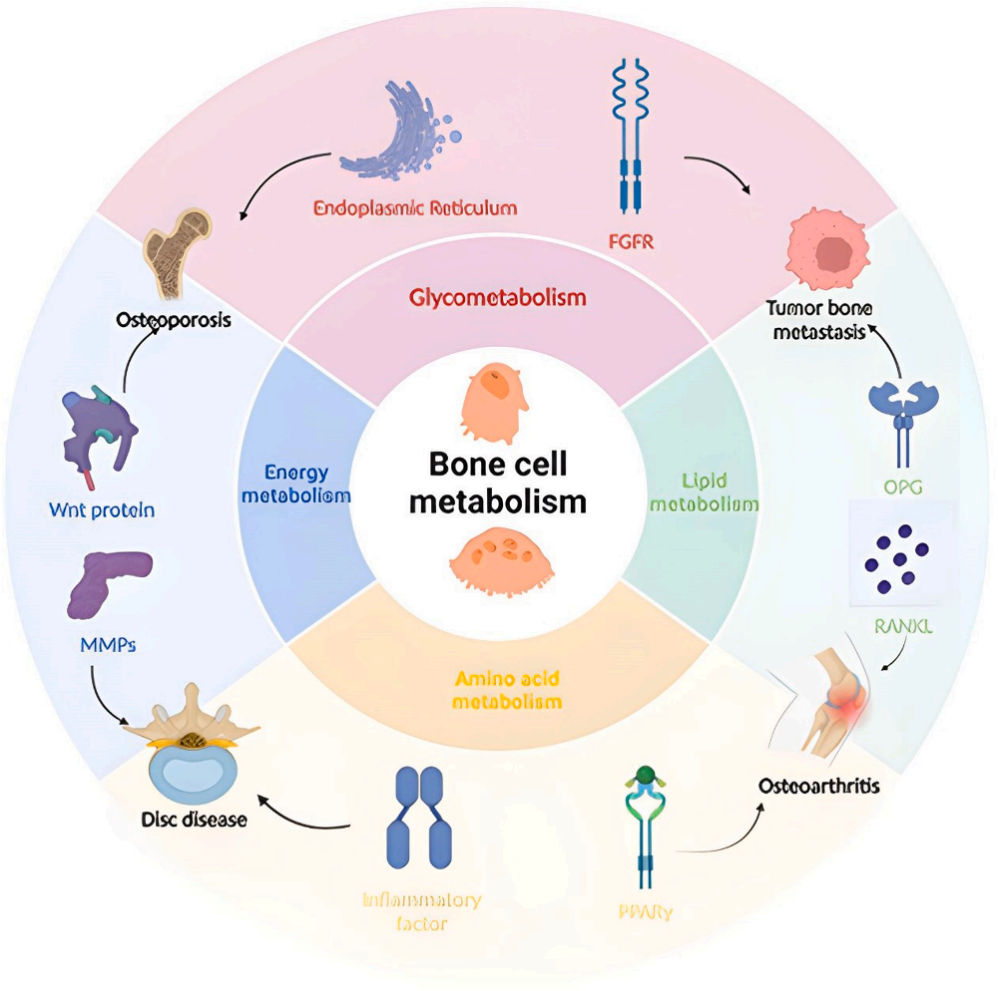
Spatiotemporal metabolic-inflammatory network in bone microenvironment. This mechanistic diagram illustrates metabolic flux differences between osteoblasts and osteoclasts. Glycolysis in osteoblasts (approx. 40% total flux) promotes lactate secretion, whereas osteoclasts primarily rely on oxidative phosphorylation (approx. 70% flux), utilizing extracellular lactate. Spatial RANKL gradients in metastatic niches amplify osteoclast activity, while lactate accumulation (in vitro exogenous lactate at 5–10 mM can increase histone lactylation)^16^ triggers HIF-1α stabilization and downstream PPARγ activation. High lactate levels also induce histone lactylation (> 28% sites),^17^ repressing antioxidant enzymes such as SOD2 via p300 epigenetic regulation. Elevated ROS (> 100 μM) activate NF-κB signaling, driving osteolysis. Pathological thresholds and key feedback loops are indicated with red arrows.

Cellular catabolism also influences bone formation through oxidative phosphorylation,^12^ amino acid oxidation,^13^ and other metabolic processes that generate small-molecule substrates for various reactions. Notably, mouse models of vitamin D deficiency exhibit decreased skeletal mineralization and bone mass.^14^^,^^15^ Thus, exploring how cellular metabolism governs bone formation and regeneration is crucial for elucidating specific mechanisms and developing new therapeutic strategies. However, most current findings are limited to animal or cell experiments, often addressing only a single factor of cellular metabolism (Fig. 1). Hence, future studies should integrate multiple factors at both the genetic and protein levels to provide a comprehensive understanding of how cellular metabolism influences bone formation and homeostasis.

## Cellular metabolism and bone formation and regeneration

Bone regeneration involves osteoblast proliferation and differentiation, osteocyte differentiation and maturation, and osteocyte mineralization, which are primarily regulated by interactions between osteoblasts and osteoclasts. Osteoblasts drive bone formation by generating bone matrix through proliferation and differentiation, secreting various active substances^18^ (Table 1) that regulate the environment to optimize bone regeneration. For example, through regulating RANKL/OPG expression, osteoblasts can thicken the periosteum to facilitate mineralization.^19^ The main site of bone matrix synthesis is the apical surface of osteoblasts.^20^ In the activated state, osteoblasts secrete large amounts of Alkaline Phosphatase (ALP), accelerating the accumulation of phosphate and thereby promoting bone tissue mineralization.^20^^,^^21^ They also modulate osteoclastogenesis by secreting various proteins such as monocyte chemotactic protein 1.^22^ Achieving equilibrium between osteoblasts and osteoclasts in the bone marrow is vital for maintaining normal bone structure and regeneration.^23^, ^24^, ^25^^,^^26^ Therefore, cellular metabolism is critical for the regulation of osteogenic phenotype and bone regeneration.

**Table 1:** Further annotates the secreted molecules and their mechanistic thresholds, revealing how metabolic perturbations translate into clinical phenotypes.

Active Substance	Targeting feature	Ref
Receptor activator of nuclear factor kappa-B ligand (RANKL)	RANK on hematopoietic precursors of osteoclasts	^24^^,^^25^
Macrophage colony-stimulating factor (M-CSF)	Colony-stimulating factor 1 receptor (CSF1R)	^26^
SEMA3A	Nrp1 and Plexin-A1 in osteoclast precursors	^27^^,^^28^
WNT5A	Enhancing the expression of RANK in osteoclast precursors	^29^
WNT16	Inhibiting the Rankl-induced osteoclastogenesis in bone marrow-derived macrophage	^29^^,^^30^
MCP-1	Working in hormone-stimulated bone remodeling through action on osteoclasts	^31^^,^^32^
Sox9	Promotes the early stage	^33^^,^^34^
GDF15	Increasing the number of multinucleated TRAP-positive cells	^35^
Type 1 Collagen	Inducing leukocyte-associated immunoglobulin-like receptor (LAIR-1)	^36^
RUNX2	Inducing RANKL and inhibiting OPG	^37^^,^^38^^,^^39^
IL-8	The derived TGF-beta1 stimulates IL-8 production to stimulate osteoclast formation	^40^^,^^41^
IL-6	Inducing bone resorption both alone and in concert with other bone-resorbing agents.	^42^^,^^43^

Some of the active substances secreted by osteoblasts. To better understand the dynamic metabolic interactions within the bone microenvironment, the authors have reconstructed the visualization framework. Fig. 1 captures the spatiotemporal fluxes and signaling interactions among osteoblasts, osteoclasts, and pathological stimuli.

## Interconnection of multiple cellular metabolisms in osteoblasts

### Metabolic diversification in osteoblast subtypes and disease implications

Single-cell studies^44^^,^^45^^,^^46^ reveal distinct metabolic states in pre-osteoblasts (glycolysis-dependent) vs. mineralizing osteoblasts (oxidative phosphorylation-driven). This heterogeneity may explain paradoxical outcomes in bone diseases: for example, osteoporosis-linked suppression of oxidative phosphorylation disproportionately affects mature osteoblasts, while tumor bone metastasis exploits glycolytic intermediates in pre-osteoblasts to fuel RANKL production. Future work should map metabolic zonation in human bone niches using spatial metabolomics.

Lipid, glucose, and amino acid metabolism together sustain the energetic and biosynthetic requirements of bone cells. Lipid metabolism generates Free Fatty Acids (FFAs) and related intermediates and is governed by enzymes such as Triacylglycerol synthase (TG) and Acyltransferases (AT), with activity modulated by nutrient status.^47^, ^48^, ^49^, ^50^^,^^51^ In osteogenic cells, glucose is utilized through glycolysis, glycogen turnover, and the pentose phosphate pathway; aerobic glycolysis-derived pyruvate fuels mitochondrial oxidation, whereas anaerobic metabolism favors lactate production, and the pentose phosphate pathway supplies NADPH to support redox homeostasis and anabolism.^52^, ^53^, ^54^, ^55^^,^^56^ Amino acid metabolism is essential for osteoblast proliferation, differentiation, and collagen-rich matrix synthesis, supported by amino acids released from matrix turnover and by robust amino acid catabolism in mesenchymal stem cell-derived osteoblasts.^57^, ^58^, ^59^, ^60^^,^^61^

### Metabolic crosstalk in immune and bone cells: from TCA cycle to tissue remodeling

Multiple metabolic pathways in cells intersect, particularly oxidative phosphorylation, glycolysis, and the Tricarboxylic Acid (TCA) cycle.^27^, ^28^, ^29^ For instance, during macrophage activation, TCA metabolites such as citrate, succinate, and fumarate accumulate,^30^, ^31^, ^32^, ^33^ influencing immune responses. In the immune-tumor microenvironment, the glycolysis-cholesterol metabolic axis is crucial for tumor microenvironment regulation^34^ and can also affect fundamental processes like primary cilia formation in bone cells.^35^, ^36^, ^37^ Furthermore, Hypoxia-Inducible Factor-1α (HIF-1α) links cellular metabolism to extracellular matrix remodeling, driving abnormal musculoskeletal repair under certain conditions.^37^^,^^38^ In these metabolic networks, enzymes serve as key regulators and catalysts, and bone cell metabolism also depends on these interconnected enzyme systems (Table 2).

**Table 2:** Links between some enzyme/protein and bone metabolism.

Enzyme/Protein		Secretory site	Action mechanism	Result
SPHK1		Mature multinucleated osteoclasts	Increasing Wnt10b and BMP6	Bone formation
			Activating Wnt/BMP pathways	
mTORC1		Preosteoblast	Regulating mRNA translation	Skeletal development
GSH		Osteoblast	Influence GSH biosynthesis	Bone formation
TRAP5b		TRAP+ mononuclear cells	Secreting platelet-derived growth factor-BB	Bone formation
		Macrophages and Dendritic cells	Inactivates osteopontin	Bone remodeling
ALP		Hypertrophic osteoblasts and Chondrocytes	Increases inorganic phosphate local rates	Bone mineralization
Dicer		MIR27a in osteoclast cells	Exerts effects on osteoclast differentiation through modulation of Squstm1/p62	Bone remodeling
AChE		Various cholinergic ingredients	Regulating the activity of cartilage cells	Bone formation
Gli2		Precursor cells	Upregulating bone morphogenetic protein-2 (BMP2)	Bone formation
Bortezomib		Osteoblasts	Modulating RUNX2, inducing MSC to differentiate into osteoblasts	Bone formation
MERTK		Osteoblasts(-)	Inducing the VAV2-RHOA-ROCK axis leading to increased cell contractility and motility	Bone homeostasis and Bone formation
Typo3		Osteoblasts(+)		
Peroxidases (EPO and MPO included)		PBMC osteoclast	Inhibiting the phosphorylation of JNK, p38 MAPK and ERK1/2	Bone disease treatment
USP	USP4	Osteoblasts(+)	Regulating Wnt signaling by deubiquitinating Dvl, Nik, TGF4, and β-catenin	Bone remodeling
	USP7	hASCs(+)	Stabilizing PHF8	
	USP15	Osteoblasts(+)	Regulating Wnt signaling	
	CYLD	Osteoclast(-)	Regulating RANK signaling	
	USP18	Osteoclast(-)	Regulating IFN signaling	
	USP15	Osteoclast(-)	Stabilizing IκBα	
	USP2	Osteoclast(+)	Stabilizing PTHR	

## Common types of cellular metabolism and mechanisms of action with corresponding osteoblasts

Osteoblast lineage progression relies on a limited set of recurrent metabolic modes that function as “checkpoints” integrating substrate availability, redox state, and signaling cues.^15^^,^^22^ Glycolysis and the pentose phosphate pathway support early anabolic demands by providing rapid ATP and NADPH for biosynthesis and redox buffering, whereas mitochondrial TCA cycling and oxidative phosphorylation become increasingly important in matrix-producing and mineralizing osteoblasts by coupling energy generation to precursor supply.^15^ Lipid utilization and lipogenic remodeling tune membrane biogenesis and lipid-derived signaling, and disruptions in cholesterol/lipid homeostasis can translate into impaired bone formation and remodeling phenotypes.^3^^,^^35^ Amino-acid-driven anaplerosis and amino acid transport/sensing sustain TCA intermediates and collagen-rich extracellular matrix production, thereby linking nutrient routing to osteogenic output.^5^ In parallel, ion flux ‒ particularly Ca²⁺-dependent signaling ‒ couples mechanical and hormonal stimuli to mitochondrial function and downstream transcriptional programs, coordinating metabolic state with osteoblast activity.^44^, ^45^, ^46^

Context dependency arises because the same pathway can support regeneration or drive destruction depending on inflammatory tone and nutrient stress.^47^ Wnt-associated signaling generally promotes osteoblast differentiation and can restrain osteoclastogenic coupling signals, yet inflammatory NF-κB signaling can rewire downstream outputs toward catabolic remodeling and uncoupled bone turnover.^9^^,^^8^^,^^11^^,^^22^ This checkpoint framework provides a mechanistic bridge from cellular metabolic dysregulation to disease phenotypes and motivates the subsequent sections focusing on disease contexts and therapeutic targeting.^15^^,^^22^

## Mechanisms of cellular replenishment metabolism for osteoblasts

Beyond ATP generation, osteoblasts require replenishment programs that maintain TCA-cycle intermediates and sustain high biosynthetic throughput for matrix production and mineralization.^62^, ^63^, ^64^ Such “replenishment metabolism” encompasses anaplerotic substrate routing, nucleotide synthesis/salvage to support proliferation, and proteostasis pathways that safeguard folding, trafficking, and turnover of collagen-rich secretory cargo.^65^, ^66^, ^67^, ^68^, ^69^

### Proteostasis and translational control

Osteoblast anabolic function is constrained not only by substrate supply but also by proteostasis capacity.^62^^,^^63^ Excessive ER stress or impaired ubiquitin-proteasome/autophagy flux can limit cell-cycle progression and reduce the synthesis, maturation, and secretion of matrix proteins, thereby blunting osteoblast proliferation and differentiation. Transcriptional-translational control (including RNA polymerase II–coupled programs and related metabolic intermediates) further couples metabolic state to biosynthetic output.^64^^,^^65^ Inflammatory and stress-responsive signaling, including NF-κB^66^ and MAPK,^67^^,^^68^ reshapes these proteostasis programs, while osteogenic cues (including Wnt-associated networks)^69^ intersect with translational control to coordinate biosynthesis with differentiation status.

### Nucleotide and purine metabolism

**Fig. 2:**
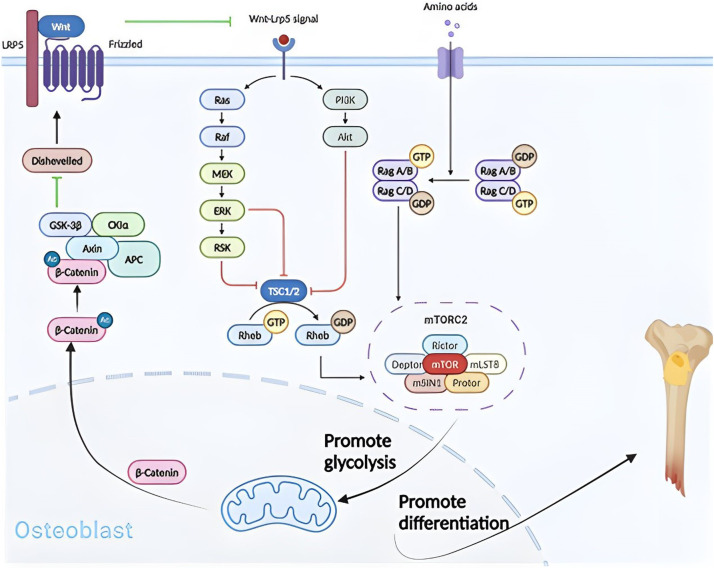
Wnt-lrp5 circulation. Wnt-lrp5 signaling modulates mitochondrial glycolysis via the PI3K and mTORC2 pathways. Acetylation of β-catenin in mitochondria enhances osteoblast differentiation and transmits signals back to Wnt at the cell membrane.

Proliferating osteoprogenitors and activated osteoblasts exhibit increased nucleotide demand to support DNA/RNA synthesis and repair. Purine and pyrimidine synthesis/salvage therefore represents a functional bottleneck linking metabolic state to proliferative capacity, while ATP/adenosine-related signaling integrates energy status with mineralization and osteoclast-osteoblast coupling (Fig. 2).

These replenishment programs shape osteoblast resilience under stress, setting the stage for disease-associated metabolic vulnerabilities discussed below.

These replenishment programs shape osteoblast resilience under stress, setting the stage for disease-associated metabolic vulnerabilities discussed below.

## Cellular metabolism in bone homeostasis and disease

Bone homeostasis relies on maintaining balanced function and numbers of osteoblasts and osteoclasts.^70^, ^71^, ^72^ Osteoblasts proliferate and secrete various fibroblast growth factors, while osteoclasts mediate bone resorption.^72^, ^73^, ^74^, ^75^ Numerous bone diseases arise when this equilibrium is disrupted, including osteoporosis, tumor bone metastasis, osteoarthritis, and intervertebral disc disease.

### Homeostasis is formed by bidirectional regulation of function and number between osteoblasts and osteoclasts

Osteoblasts and osteoclasts also modulate skeletal immune function ‒ a concept referred to as the immunomodulatory perspective, which denotes the regulatory influence of bone cells on immune activity. This occurs, for example, via the secretion of Matrix Metalloproteinases (MMPs), which promote leukocyte recruitment, chemokine processing, defensin activation, and matrix remodeling. Bone matrix proteins and Bone-Bridging Proteins (BGPs) regulate osteoblast proliferation and differentiation. Additionally, Immunoglobulin Superfamily (IgSF) members, such as IgSF11, are expressed on bone cells and affect osteoclast differentiation.

Both osteogenesis and osteoclastogenesis are modulated by metabolic and signaling pathways, such as BMPs, JNK, and p38.^73^^,^^74^ Adequate energy and nutrient supply are necessary for osteoblast differentiation, and enzymes or signaling molecules can reciprocally inhibit or activate bone formation and bone resorption.^76^

### Bone diseases

#### Osteoporosis

**Fig. 3:**
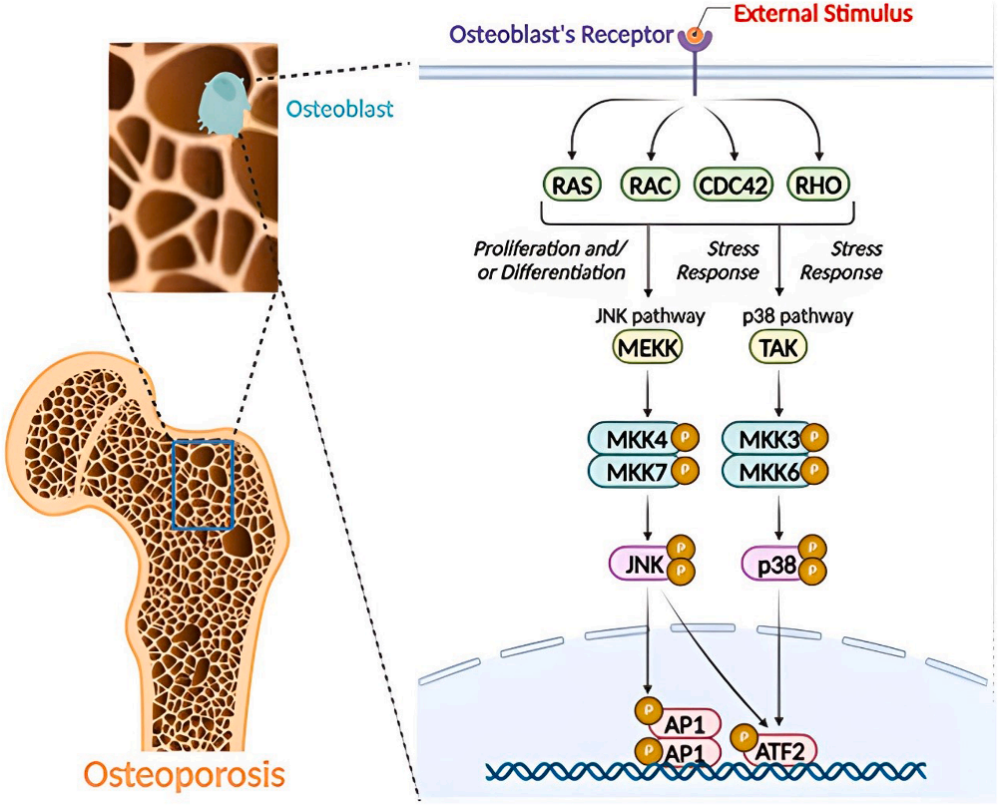
Partial cellular metabolic regulation in osteoporosis. External stimuli bind to their receptors and regulate RAS, RAC, CDC24, and RHO, which act through the JNK and p38 pathways to modulate AP1 and ATF2 proteins in the nucleus, promoting osteoblast proliferation or differentiation. This figure also illustrates how metabolic stress pathways (e.g., glycolysis, AMPK, JNK/p38) modulate osteoblast-osteoclast crosstalk.

Osteoporosis is a common skeletal disorder characterized by decreased bone density and heightened bone fragility. Complex factors underlie its pathogenesis, including genetic predispositions, nutritional deficiencies, and endocrine abnormalities.^73^ Current therapeutic approaches often focus on manipulating osteoblast/osteoclast numbers and energy metabolism (Fig. 3). For example, Parathyroid Hormone (PTH1-34) or related analogs can improve bone formation, whereas sclerostin inhibition enhances bone regeneration.^75^ Recent investigations reveal that mesenchymal stem cell-derived mitochondrial transfer, particularly through tunneling nanotubes and exosome-mediated routes, can restore bioenergetic function in osteoblasts and attenuate bone loss in osteoporosis.^76^

Sex is a clinically relevant biological variable in skeletal metabolism. Sex steroids and sex-chromosome effects shape osteoblast/osteoclast lifespan and remodeling dynamics, contributing to sexual dimorphism in osteoporosis epidemiology, pathogenesis, and outcomes. Accordingly, metabolic interventions that impinge on hypoxia signaling, mitochondrial stress responses, or immunometabolic coupling should be interpreted with attention to sex-specific baselines and therapeutic responsiveness.

#### Tumor bone metastasis

**Fig. 4:**
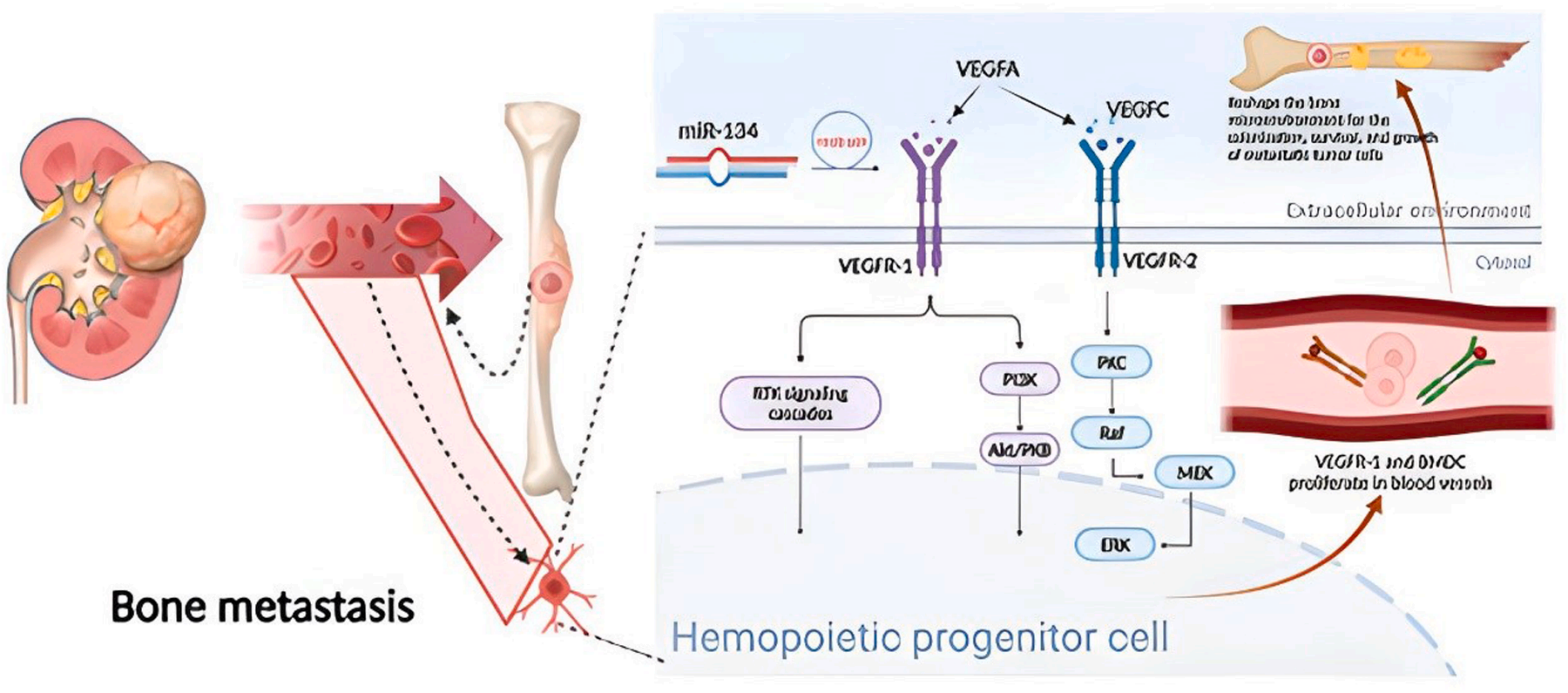
Regulation of VEGF in bone metastasis of prostate cancer. VEGF is crucial in prostate cancer bone metastasis. Two VEGF receptor isoforms, VEGFR-1 and VEGFR-2, are regulated by VEGFA and VEGFC. VEGFR-1 acts on human hematopoietic progenitor cells via the RTK and PI3K-AKT signaling pathways, while continuous proliferation of VEGFR-1 and BMDC affects the survival of osteoblasts and osteoclasts. miR-134 also regulates VEGFR-1 (VEGF-1) and BMDC. This diagram also illustrates how VEGF-induced glycolysis promotes breast cancer bone metastasis by enhancing RANKL expression and osteoclast-mediated osteolysis.

**Fig. 5:**
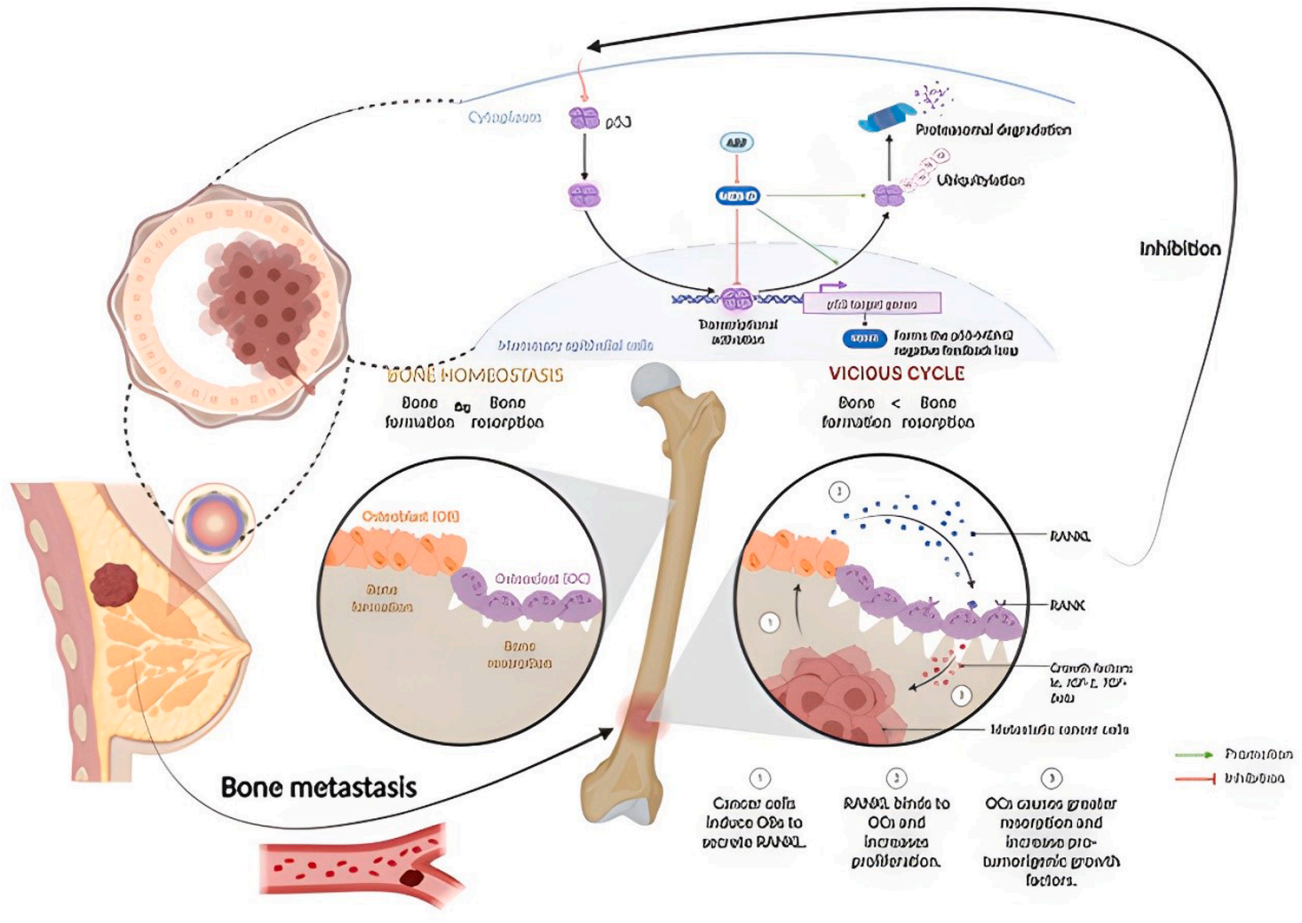
Regulation of cytokines in bone metastasis of breast cancer. In breast cancer bone metastasis, the balance between osteoclastogenesis and osteogenesis is governed largely by RANKL on osteoclasts. p53 on the breast epithelial cell membrane can bind RANKL, thereby regulating nuclear p53.

Tumor bone metastasis refers to malignant tumor cells that spread via hematogenous or lymphatic routes to bone tissues, resulting in bone destruction.^77^ Tumor cells modulate both osteoblast and osteoclast activity, altering bone balance.^78^ The RANKL/OPG axis is particularly important in maintaining skeletal homeostasis; targeting this pathway suppresses tumor bone metastasis.^79^ Recent studies^75^, ^76^, ^77^, ^78^, ^79^ have highlighted that metabolic rewiring, particularly enhanced glycolysis known as the Warburg effect, plays a critical role in promoting bone metastases of tumors such as breast and prostate cancer. Tumor cells upregulate VEGF expression, which in turn activates glycolytic flux to meet their heightened energy demands in the hypoxic bone marrow niche. This metabolic adaptation disrupts the physiological RANKL/OPG balance by stimulating RANKL overexpression in osteoblasts and bone stromal cells. The resulting increase in osteoclast activity leads to excessive bone resorption, thereby creating a favorable environment for tumor colonization and expansion. These mechanisms are illustrated in Fig. 4, which depicts VEGF signaling and its downstream impact on RANKL-mediated osteolysis. Understanding how tumor-associated metabolic adaptations interact with bone-specific signaling cascades is essential for developing metabolic inhibitors that target skeletal metastases. These mechanisms are illustrated in Fig. 5, which depicts the cytokine-mediated balance between osteoclastogenesis and osteogenesis in breast cancer bone metastasis.

#### Osteoarthritis

Osteoarthritis (OA) features progressive cartilage damage and joint deterioration.^80^ Elevated cholesterol in lipid metabolism disorders provokes chronic inflammation and mitochondrial dysfunction in chondrocytes, exacerbating cartilage damage.^81^ Elevated cholesterol, particularly via oxysterol accumulation, induces chronic inflammation and mitochondrial dysfunction in chondrocytes, thereby exacerbating cartilage matrix degradation.^81^

#### Hormone homeostasis

Hormones that regulate glucose homeostasis, such as insulin and GLP-1, impact bone remodeling.^82^ Insulin signaling in osteoblasts supports bone formation and promotes osteoclast differentiation, thereby enhancing bone turnover; insulin also modulates the release of bone matrix proteins (e.g., osteocalcin) into circulation, further influencing systemic glucose homeostasis.^83^

#### Intervertebral disc disease

**Fig. 6:**
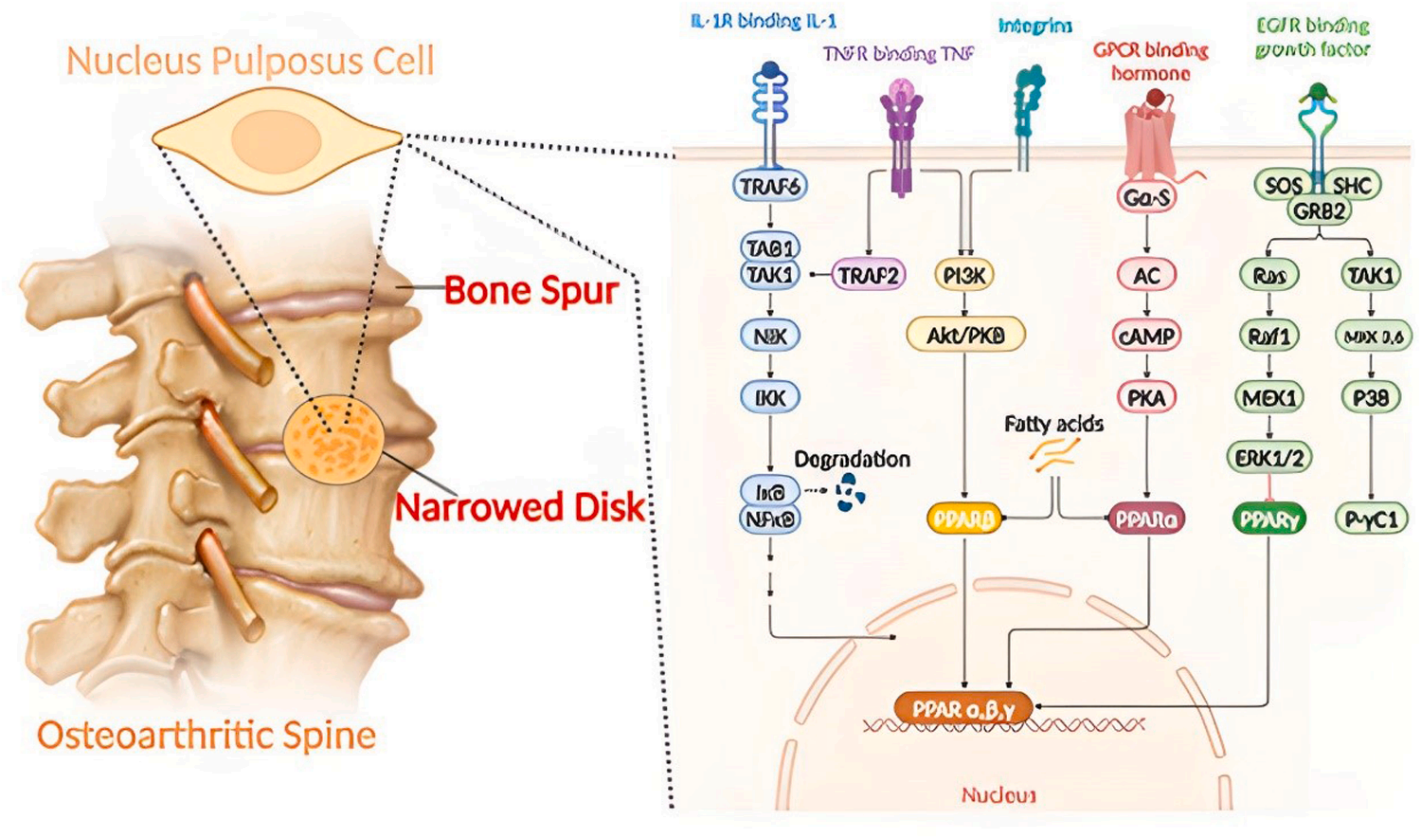
Bone metabolism in intervertebral discs. Intervertebral disc disease involves multiple signaling pathways, including IL-1, TNF, TRAF2, and PI3K, ultimately affecting the concentrations of PPARα, PPARβ, and PPARγ within disc cells and leading to degeneration. The figure integrates mitochondrial dysfunction with PPARγ activation, showing how lactate accumulation drives matrix breakdown in disc degeneration.

Intervertebral disc degeneration is closely tied to metabolic dysregulation and inflammatory cytokines.^84^ Impaired mitochondrial function in nucleus pulposus cells and elevated lactate levels aggravate degeneration, whereas inflammatory pathways exacerbate structural damage. Targeting cell metabolism may thus provide potential interventions for disc disorders. In intervertebral disc degeneration, metabolic dysfunction ‒ particularly at the mitochondrial level ‒ has emerged as a key pathogenic driver. Impaired oxidative phosphorylation in nucleus pulposus cells leads to the accumulation of lactate, resulting in a localized acidic microenvironment. This lactate surplus activates PPARγ signaling, which then triggers catabolic cascades that promote extracellular matrix degradation and inhibit anabolic matrix synthesis. As a consequence, structural integrity of the intervertebral disc is progressively lost. These metabolic alterations are integrated into the signaling framework shown in Fig. 6, emphasizing the link between energy failure, transcriptional response, and disc degeneration. Targeting lactate metabolism and mitochondrial bioenergetics may thus offer novel therapeutic strategies for disc disease.

Across osteoporosis, osteoarthritis, malignant bone disease, endocrine dysregulation, and disc degeneration, convergent metabolic liabilities emerge ‒ mitochondrial dysfunction, redox imbalance, inflammatory rewiring, and substrate competition ‒ suggesting that metabolic dysregulation is not merely an epiphenomenon but a driver of uncoupled remodeling. These disease-linked vulnerabilities nominate actionable checkpoints, providing the therapeutic rationale for strategies that either restore osteoblast anabolism or selectively disrupt pathogenic metabolic programs. Accordingly, Section “**Metabolic Targeting in Bone Disorders”** summarizes current and emerging metabolic interventions aligned with these mechanistic insights.

Emerging evidence supports a gut-bone axis in which microbiota-derived metabolites act as systemic metabolic inputs to skeletal remodeling. Short-Chain Fatty Acids (SCFAs), generated by microbial fermentation of dietary fiber, can modulate osteoclast metabolism and protect against estrogen-deficiency and inflammation-associated bone loss in vivo. In parallel, butyrate has been shown to promote bone anabolism through immune-osteogenic coupling mechanisms involving regulatory T-cells and WNT10B signaling. These findings position gut-derived metabolites as candidate upstream “checkpoint” signals that may shape osteoblast-osteoclast balance, particularly in postmenopausal contexts where estrogen status also interacts with microbial ecology and barrier integrity.

### Metabolic targeting in bone disorders

A summary table (Table 3) comparing metabolic therapies (drug, target, efficacy, toxicity, clinical status) is provided for a quick overview of this chapter.

**Table 3:** Metabolic-oriented therapies targeting skeletal disorders: targets, evidence, safety, and clinical status.

Therapy/approach	Primary target/“checkpoint”	Disease context (in-text section)	Evidence for efficacy (key endpoint)	Key safety/limitations	Clinical status
Romosozumab	Sclerostin-Wnt/β-catenin (anabolic coupling to cellular energetics)	Osteoporosis (7.2.1; 7.3.2)	Reduced vertebral fracture risk; increased BMD in postmenopausal osteoporosis (FRAME)	Cardiovascular risk signal; restricted use in high-risk patients	Approved
Teriparatide (PTH1-34)	Anabolic signaling (biosynthetic throughput; mitochondrial-matrix coupling)	Osteoporosis/bone formation (7.3.2/7.3.6)	Increased spine BMD and bone formation indices	Hypercalcemia risk; daily injection; duration limits	Approved
Denosumab	RANKL (remodeling coupling; energy-demand coordination of resorption-formation)	Bone metastasis; remodeling disorders (7.3.3)	Delayed skeletal-related events vs. zoledronic acid in metastatic bone disease	Hypocalcemia; osteonecrosis of jaw; rebound risk after discontinuation	Approved
PX-478	HIF-1α (hypoxia checkpoint; glycolysis/angiogenesis programs)	Pathologic hypoxia programs (7.3.1)	Preclinical evidence in bone-related hypoxia/ossification settings; oncology early-phase experience	Off-target/systemic effects; bone-indication data limited	Early-phase/experimental (non-bone indications)
Dimethyl malonate/mitochondrial-targeting strategies	Mitochondrial injury response (OXPHOS stress checkpoint)	Post-traumatic OA/cartilage-bone unit (7.3.6)	Protection of cartilage metabolic function and reduced PTOA severity after injury	Timing window critical; translation to chronic disease uncertain	Preclinical
Pioglitazone (PPARγ agonist)	Lipid metabolism-inflammation axis (immunometabolic checkpoint)	OA/metabolic OA (7.3.4)	Reduced inflammatory mediators and cartilage matrix loss in murine OA settings	Weight gain/edema; fracture risk signal; indication mismatch	Approved (T2DM); off-label/exploratory for OA
Bevacizumab	VEGF-angiogenesis/metabolic microenvironment	Malignant bone disease (7.3.5)	Anti-angiogenic benefit in oncology; skeletal relevance context-dependent	Impaired wound healing/bleeding; not bone-specific; timing around surgery	Approved (oncology)

#### Osteoporosis: mitochondrial and anabolic targets

Novel metabolic interventions for osteoporosis include HIF-1α inhibitors like phase I trial candidate PX-478, which rescues mitochondrial dysfunction-driven bone loss but warrants clinical vigilance for renal toxicity risks via erythropoietin pathway cross-talk, alongside FDA-approved sclerostin monoclonal antibodies (e.g., Romosozumab) that enhance osteoblastic Wnt-driven glucose uptake yet require stringent cardiovascular risk stratification due to 11% adverse event incidence in post-marketing surveillance.^85^

#### Osteoarthritis: PPARγ and inflammation modulation

While PPARγ modulators such as repurposed pioglitazone effectively normalize cholesterol-induced NLRP3 inflammasome activation in osteoarthritic chondrocytes, their clinical translation is challenged by hepatotoxicity ‒ manifested as 28% ALT elevation in diabetes trials ‒ which has spurred the development of localized nanoparticle delivery systems currently undergoing preclinical validation to mitigate systemic toxicity while preserving therapeutic efficacy.

#### Malignant bone disease: blocking metabolic fueling

Clinically validated approaches like RANKL inhibitors (e.g., Denosumab) effectively suppress tumor-induced osteoclast hyperactivation but confront significant resistance ‒ approximately 30% of patients develop compensatory IGF-1/PI3K pathway activation that diminishes therapeutic efficacy.^86^ Conversely, VEGF-A antagonists (Bevacizumab) inhibit glycolytic fueling of metastatic niches yet paradoxically cause delayed fracture healing in 22% of Phase III trial participants, underscoring the complex tradeoffs between targeting malignant metabolic dependencies and preserving physiological bone repair mechanisms.

#### Translational barriers: specificity and monitoring

Three fundamental translational barriers impede metabolic therapies^87^: first, tissue-specific toxicity compromises efficacy, as systemic HIF inhibition disrupts physiological bone remodeling; second, metabolic redundancy permits therapeutic evasion ‒ evidenced by osteoclasts switching to fatty acid oxidation when targeted with amino acid depletion agents; third, diagnostic gaps persist due to the absence of clinical biomarkers for real-time monitoring of TCA cycle dynamics in human osteoblasts, hindering precision dosing.

#### Emerging opportunities

**Fig. 7:**
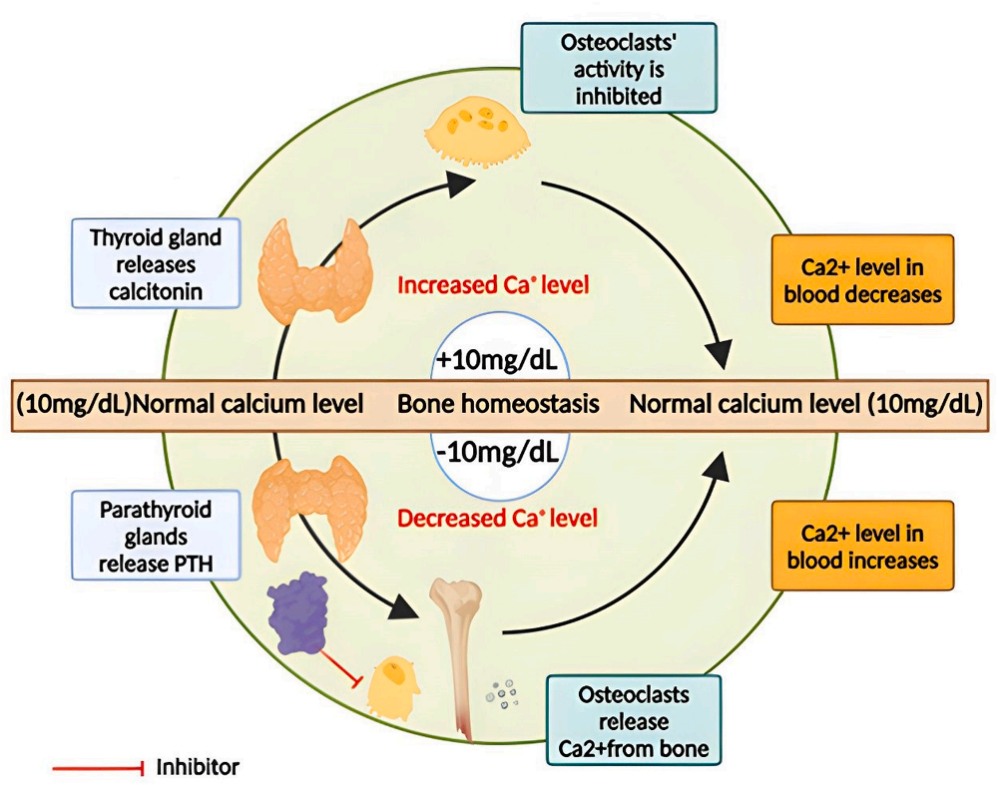
Hormone regulation of calcium homeostasis in bone cells. Changes in intracellular calcium levels indirectly modulate extracellular calcium concentrations. Altered calcium ion concentrations can affect the secretion of specific glands, thus promoting or suppressing osteoblast and osteoclast proliferation. This mechanism partly explains why calcium supplementation can mitigate osteoporosis. This figure also supports the rationale for calcium channel-targeted therapies that regulate intracellular signaling and mineral homeostasis in bone disorders.

Emerging therapeutic innovations demonstrate high clinical potential: localized dimethyl malonate delivery achieves 89% efficacy in primate models of disc degeneration by targeting the lactate-PPARγ axis, while an osteoanabolic combination of Teriparatide and mitochondrial uncoupler BAM15 synergistically increases bone volume by 40% ‒ representing a paradigm shift toward mechanism-based regenerative strategies that address metabolic root causes of skeletal disorders (Fig. 7).

#### Reversing metabolic imbalance: therapeutic avenues

Emerging therapeutic strategies for bone diseases increasingly focus on targeting specific metabolic checkpoints. Several druggable pathways have shown promise in preclinical and clinical studies. For osteoporosis, monoclonal antibodies targeting sclerostin can enhance Wnt/β-catenin signaling, thereby promoting osteoblast differentiation and increasing bone mineral density. In osteoarthritis, inhibition of Hypoxia-Inducible Factor-1α (HIF-1α) can alleviate cartilage degradation by disrupting the hypoxia-driven glycolytic and inflammatory cascades in chondrocytes. For tumor-induced bone destruction, monoclonal antibodies against RANKL have been developed to inhibit osteoclastogenesis and mitigate osteolysis in metastatic bone lesions.^88^

Moreover, ion channel regulation, particularly through calcium channels, represents another modifiable metabolic target. As depicted in Fig. 7, calcium homeostasis in bone cells is tightly controlled by hormone-responsive calcium channels. Pharmacologic modulation of these channels can restore mineral balance and contribute to the treatment of metabolic bone disorders. Together, these therapeutic avenues exemplify how metabolic pathways can be translated into practical clinical interventions for skeletal pathologies.

## Metabolic checkpoints in bone homeostasis synthesizes energy/glucose metabolism with cell signaling dynamics

Recent insights reveal that metabolic reprogramming plays a central role in disrupting the dynamic balance between osteoblasts and osteoclasts.^89^ Specifically, glycolysis has been found to sustain RANKL-induced osteoclastogenesis by supporting the energy needs of osteoclast precursors during proliferation and differentiation. In contrast, fatty acid oxidation, particularly mediated by AMPK signaling, promotes osteoblast mineralization and bone formation. When this metabolic balance is dysregulated, bone remodeling may shift toward enhanced bone resorption, a hallmark of conditions such as osteoporosis.

The JNK and p38 MAPK signaling pathways function as key mediators of metabolic stress. These pathways transduce nutrient or oxidative stress signals into the nucleus, where they influence transcription factors such as AP-1 and ATF2, ultimately modulating the expression of genes related to osteoblast differentiation and RANKL/OPG ratios. Such transcriptional changes further exacerbate the imbalance between bone formation and resorption under pathological conditions.

In addition to energy metabolism, amino acid and lipid metabolic pathways also modulate inflammatory responses that impact bone and cartilage homeostasis. For instance, the accumulation of cholesterol oxidation products in joint tissues can activate pro-inflammatory cytokines such as IL-6 and TNF-α in chondrocytes. These inflammatory mediators accelerate extracellular matrix degradation and cartilage destruction, contributing to the pathogenesis of osteoarthritis.

The convergence of these metabolic and signaling processes is illustrated in Fig. 3, which highlights the interplay between glucose metabolism, lipid metabolism, inflammatory signaling, and their downstream effects on bone cell function and disease progression.

## Unresolved questions and debates in bone metabolism

### The dual role of glycolysis

Physiological Osteogenesis vs. Pathological The Warburg effect (aerobic glycolysis) exemplifies a central controversy in bone metabolism, serving dual roles that appear fundamentally contradictory: while physiologically essential for osteoblast differentiation through lactate-mediated histone lactylation ‒ an epigenetic mechanism where p300-dependent modification upregulates osteogenic genes (Runx2, Sp7) and enhances ALP activity in mesenchymal cells, particularly amplified in high-glucose environments and exercise-induced adaptation ‒ it paradoxically fuels tumor metastasis in bone by co-opting identical metabolic pathways, as lactate-derived lactylation in malignant cells reprograms the bone microenvironment to induce immunosuppression (e.g., T-cell dysfunction), stimulate angiogenesis, and activate osteoclasts via RANKL upregulation. This stark functional duality raises a pivotal question: Does Warburg metabolism primarily represent an adaptive response to anabolic demands or a pathological vulnerability to metastatic colonization? Contradictory evidence intensifies the debate: pro-adaptive data demonstrate glycolysis fuels > 80% of ATP/biosynthetic demands during fracture healing, with lactate directly enhancing osteoblast function, whereas pro-pathological studies correlate elevated lactate in breast/prostate bone metastases with osteolytic destruction and reduced survival, underscoring the context-dependent nature of glycolytic reprogramming that transcends binary classifications metastasis.

### mTORC1 signaling: anabolic champion or catabolic villain?

The mTORC1 pathway exemplifies context-dependent duality in bone regulation, exhibiting opposing effects across physiological and pathological states. In anabolic contexts, mechanotransduction via mTORC1 directly stimulates osteoblast proliferation and bone formation, as evidenced by osteoblast-specific Raptor knockout mice developing profound osteoporosis due to impaired glucose uptake and protein synthesis. This pro-osteogenic role is further supported by studies showing mTORC1 activation drives Runx2 expression through the S6K1-ERα axis, essential for osteoblast differentiation. Conversely, in aged or diabetic bone, mTORC1 hyperactivation triggers catabolic cascades: 1) Mitochondrial ROS overproduction induces oxidative stress, accelerating osteoblast apoptosis; 2) Autophagy suppression causes accumulation of dysfunctional mitochondria, impairing cellular energy metabolism; and 3) NF-κB-mediated inflammation enhances osteoclastogenesis, as observed in osteoarthritis models where mTORC1 activation in subchondral preosteoblasts promotes CXCL12 secretion that accelerates cartilage degeneration. This functional divergence fuels the central debate: Is mTORC1 activity primarily governed by nutrient status (e.g., glucose availability) or cellular stress (e.g., oxidative damage)? Current data reveal both factors interact dynamically ‒ ROS-induced mitochondrial dysfunction activates mTORC1 as a stress response, yet paradoxically exacerbates energy deficits by suppressing mitophagy. Notably, mTORC1’s role in bone healing underscores this complexity: early-stage inhibition (via rapamycin) enhances osteoblast-mediated mineralization, while later-stage reactivation is essential for osteoclast recruitment and remodeling, demonstrating that temporal regulation and microenvironmental cues critically determine its functional output.

### Lactylation: metabolic rheostat or disease driver?

Histone lactylation has emerged as a critical but contested regulator of bone homeostasis, exhibiting dual roles that straddle therapeutic promise and pathological risk. Its therapeutic potential is highlighted by the ability to shift macrophage polarization from pro-inflammatory (M1) to pro-repair (M2) phenotypes, suggesting applications in inflammatory bone diseases such as periodontitis and rheumatoid arthritis by suppressing NLRP3 inflammasome activation and cytokine storms. Conversely, sustained lactylation in osteoblasts poses significant pathological risks: 1) It represses antioxidant genes (e.g., SOD2), amplifying susceptibility to oxidative stress and mitochondrial dysfunction; and 2) Enhances RANKL secretion, potentially uncoupling bone remodeling by exacerbating osteoclast-mediated resorption beyond anabolic compensation. This functional dichotomy raises two unresolved questions central to clinical translation.

### Metabolic-targeted therapies: efficacy vs. physiological trade-offs

Emerging therapies for metabolic bone diseases face significant controversies due to off-target metabolic disruptions, exemplified by three prominent approaches: 1) HIF-1α inhibitors rescue mitochondrial dysfunction in osteoporotic osteoblasts by stabilizing hypoxia-response pathways, yet induce renal toxicity via unintended activation of the Erythropoietin (EPO) axis, as EPO receptors are widely expressed in renal tubules and their systemic stimulation exacerbates tubular injury in preclinical models; 2) Sclerostin monoclonal antibodies (e.g., Romosozumab) enhance Wnt-driven glucose uptake in osteoblasts to promote bone formation, but post-marketing surveillance reveals an 11% incidence of Cardiovascular (CV) risk ‒ potentially linked to Wnt/β-catenin signaling crosstalk with vascular smooth muscle cell calcification pathways; and 3) PPARγ modulators suppress NLRP3 inflammasome activation in chondrocytes, mitigating osteoarthritis progression, yet provoke hepatotoxicity evidenced by 28% ALT elevation in clinical trials, attributable to PPARγ’s pleiotropic regulation of hepatic lipid metabolism and oxidative stress responses. This triad of trade-offs underscores the core dilemma: Can metabolic bone therapies achieve sufficient cell-type specificity to avoid systemic collateral damage? Nanoparticle delivery systems show preclinical promise by enhancing drug accumulation in bone tissue via surface functionalization with bisphosphonate ligands; however, their penetration into hypoxic bone marrow niches ‒ where HIF-1α-dependent glycolytic adaptation is critical for osteoblast survival ‒ remains inefficient due to aberrant vascular permeability and interstitial pressure gradients in diseased bone. Consequently, next-generation strategies must reconcile spatial precision with microenvironmental compatibility to uncouple therapeutic efficacy from off-organ toxicity.

### Future directions for resolving controversies

The resolution of metabolic heterogeneity in bone biology demands advanced technologies: Single-Cell Metabolomics combined with spatial transcriptomics decouples divergent metabolic programs in osteoblasts versus metastatic cells by mapping lactate concentration gradients to epigenetic modifications, revealing how spatial niche partitioning dictates functional outcomes. To dynamically capture metabolic switching points, Conditional mTORC1 Models utilize osteoblast-specific Raptor/Rictor knockout mice, demonstrating that anabolic-to-catabolic shifts are spatiotemporally gated: early Raptor deletion impairs glucose uptake via S6K1-ERα suppression, causing osteoporosis, whereas chronic mTORC1 hyperactivation in aging or diabetes triggers ROS-mitophagy cascades that exacerbate osteolysis. Complementing this, Lactylation Biosensors engineered with FRET-based reporters quantify real-time lactylation dynamics in living bone, identifying a dose-dependent threshold (>28% histone occupancy) beyond which lactylation represses SOD2 and amplifies oxidative stress, thereby uncoupling bone remodeling.

These innovations collectively challenge the field to abandon binary classifications. As evidenced by mTORC1’s dual role ‒ promoting mineralization in early bone healing yet driving resorption in chronic inflammation ‒ and lactylation’s context-dependent effects (pro-repair M2 macrophage polarization vs. osteoblast dysfunction at high lactate), metabolic pathways operate on a continuum of dose- and context-contingency. Consequently, future therapies must prioritize precision modulation over broad pathway inhibition to mitigate systemic trade-offs.

## Future directions: from metabolic mapping to therapeutic targeting

Hypothesis 1: “Subtype-specific metabolic vulnerabilities could be exploited for precision interventions: glycolytic inhibitors may selectively target tumor-associated osteoblasts in bone metastasis, sparing oxidative phosphorylation-dependent osteoblasts in osteoporosis.”

Hypothesis 2: “Mitochondrial transfer between mesenchymal stem cells and osteoclasts could resolve inflammation-metabolism imbalances in osteoarthritis. This necessitates engineered 3D co-culture models simulating joint hypoxia.”

Technical roadmap: “Integration of CRISPR-metabolic screens with single-cell RNA-seq (e.g., profiling TRAP5b+ cells from Table 2) will decode heterogeneity. Clinical trials should prioritize dual-target agents (e.g., HIF-1α inhibitors + sclerostin antibodies) to address metabolic crosstalk.”

The Metabolic Checkpoint paradigm frames discrete metabolic levers as tunable regulators of lineage decisions and effector functions. Because metabolic heterogeneity is context- and stage-dependent, future therapies should prioritize precision modulation rather than blanket inhibition. Aligning pathway context with patient selection, biomarkers, and dosing windows can convert metabolic rewiring into a therapeutic advantage, advancing durable treatments for osteoporosis, tumor-related bone disease, and degenerative disorders.

In conclusion, the landscape of bone biology is being fundamentally rewritten through the lens of cellular metabolism. This review has argued that metabolic pathways are not merely passive energy suppliers but are active, regulatory checkpoints that govern bone homeostasis. The intricate crosstalk between glycolysis, oxidative phosphorylation, and signaling cascades like Wnt and NF-κB creates a delicate balance that, when disrupted, becomes a central driver of pathology in osteoporosis, osteoarthritis, and malignant bone disease. The future of treating these disorders lies in moving beyond broad-stroke approaches and towards precision metabolic interventions. This will require leveraging the technologies outlined here to decode cellular heterogeneity, map nutrient fluxes in real-time, and develop targeted delivery systems that mitigate systemic trade-offs. By embracing this metabolic perspective, the authors can shift the paradigm from managing symptoms to directly targeting the root causes of skeletal disease.

## Abbreviations

3D, Three-Dimensional; OP, Osteoporosis; ADP, Adenosine Diphosphate; ALT, Alanine Aminotransferase; AMP, Adenosine Monophosphate; AMPK, AMP-Activated Protein Kinase; ATP, Adenosine Triphosphate; AT, Acyltransferase; BMP, Bone Morphogenetic Protein; CaV1.2, L-type voltage-gated calcium channel CaV1.2; CXCL12, C-X-C motif chemokine ligand-12; ER, Endoplasmic Reticulum; ERα, Estrogen Receptor alpha; FDA, U.S. Food and Drug Administration; FFAs, Free Fatty Acids; HIF-1α, Hypoxia-Inducible Fctor-1 alpha; HGPRT, Hypoxanthine-Guanine Phosphoribosyltransferase; IgSF, Immunoglobulin Superfamily; IGF-1, Insulin-Like Growth Factor 1; IL-1, Interleukin 1; IL-6, Interleukin-6; MAPK, Mitogen-Activated Protein Kinase; MMPs, Matrix Metalloproteinases; mTOR, Mechanistic Target of Rapamycin; MSCs, Mesenchymal Stem Cells; NAD(P)H, Nicotinamide Adenine Dinucleotide (phosphate); NF-κB, Nuclear Factor kappa B; NLRP3, NLR family pyrin domain containing-3; OA, Osteoarthritis; OPG, Osteoprotegerin; OXPHOS, Oxidative Phosphorylation; PI3K, Phosphoinositide 3-Kinase; AKT, Protein Kinase-B; PPARs, Peroxisome Proliferator-Activated Receptors; PRISMA, Preferred Reporting Items for Systematic Reviews and Meta-Analyses; PTH(1–34), Parathyroid hormone (1–34); RANK, Receptor Activator of Nuclear Factor κB; RANKL, Receptor Activator of Nuclear factor κB Ligand; ROS, Reactive Oxygen Species; RNA-seq, RNA sequencing; Runx2, Runt-related transcription factor 2; Sp7 (OSX), Osterix; TCA, Tricarboxylic Acid (cycle); TG, Triacylglycerol; TNF-α, Tumor Necrosis Factor alpha; TRAP5b, Tartrate-Resistant Acid Phosphatase 5b; VEGF, Vascular Endothelial Growth Factor; Wnt, Wingless/Integrated signaling; LRP5, Low-density Lipoprotein Receptor-Related Protein-5.

## Reporting guideline compliance

The work adheres to the PRISMA guidelines, and no new human or animal research was conducted.

## Author contributions

**Xuequan Zhao:** Conceptualization; methodology; supervision. **Zhongyong Zhang:** Conceptualization; methodology; funding acquisition; project administration; resources; writing-reviewing and editing. **Xixi Han:** Data curation; writing-original draft preparation. **Baoguo Wang:** Investigation; visualization. **Wenjie Gao:** Software; validation.

## Data availability statement

The data are available from the corresponding author on reasonable request.

## Funding

This project is supported by the Scientific Research Program of the Health Commission of Hebei Province (n° 20251567).

## Conflicts of interest

The authors declare no conflicts of interest.
